# *Borniopsis mortoni* sp. n. (Heterodonta, Galeommatoidea, Galeommatidae *sensu lato*), a new bivalve commensal with a synaptid sea cucumber from Japan

**DOI:** 10.3897/zookeys.615.8125

**Published:** 2016-09-07

**Authors:** Ryutaro Goto, Hiroshi Ishikawa

**Affiliations:** 1 Museum of Zoology and Department of Ecology and Evolutionary Biology, University of Michigan, 1109 Geddes Avenue, Ann Arbor, Michigan 48109-1079, USA; 2 965-1 Kawachi-ko, Uwajima, Ehime 798-0075, Japan

**Keywords:** Apodida, Bivalvia, Borniopsis, Byssobornia, commensalism, Galeommatoidea, host shift, Holothuroidea, Pseudopythina, symbiosis, Synaptidae

## Abstract

The Galeommatoidea is a bivalve superfamily that exhibits high species diversity in shallow waters. Many members of this superfamily are associated commensally with burrowing marine invertebrates in benthic sediments. The genus *Borniopsis* is known only from eastern Asia and exhibits high host diversity (e.g., mantis shrimps, crabs, holothurians, sipunculans and echiurans). A new species, *Borniopsis mortoni* **sp. n.**, is described from mud flats at the mouth of the Souzu River, southwestern Shikoku Island, Japan. This species has elongate-ovate shells covered by a tan to dark brown periostracum, and lives attached by both its foot and byssal threads to the body surface of the synaptid sea cucumber *Patinapta ooplax*. Several individuals of *Borniopsis mortoni* are often found on the same host, but sometimes more than 10 individuals can occur together. *Borniopsis mortoni* is one of the smallest species in this genus. Probably, its small body size is an adaptation to the mode of life in a narrow host burrow. Until now, only two other *Borniopsis* species were known to have commensal associations with synaptids. Thus, this is the third example of a synaptid-associated species from this genus. In addition, we briefly review the galeommatoideans commensal with apodid sea cucumbers.

## Introduction

The Galeommatoidea is a superfamily of small bivalves that exhibits tremendous diversity in the intertidal zone (Bouchet et al. 2002, Paulay 2003, Lützen and Nielsen 2005). Many members of this superfamily are commensals associated in highly specific relationships with benthic invertebrates that burrow in soft sediments (Boss 1965, Morton and Scott 1989, Li et al. 2012, Goto et al. 2012). Most commensal galeommatoideans live attached directly onto the host’s body surface or the walls of its burrow (Boss 1965, Morton and Scott 1989, Goto et al. 2012).

The genus *Borniopsis* was established for *Borniopsis tsurumaru* Habe, 1959 as the type species with a second species, *Borniopsis ariakensis* Habe, 1959, from Japan (Habe 1959). Morton and Scott (1989) described four Pseudopythina (Borniopsis) species from Hong Kong. After this publication, *Borniopsis* was frequently synonymized with *Pseudopythina* (e.g., Lützen et al. 2004, Goto et al. 2012). However, Huber (2015) noticed that these East Asian *Pseudopythina* species are distinct from the type species *Pseudopythina macandrewi* (P. Fischer, 1867), which occurs only in the eastern Atlantic Ocean, in dentition, seminal receptacles, and demibranchs and thus he transferred them to *Borniopsis* together with some species of *Byssobornia*, *Squillaconcha* and *Kellia* (Huber, 2015), although *Borniopsis fujitaniana* (Yokoyama, 1927) was recently reassigned to the genus *Tellimya* (Goto et al., 2016). As a result, at least nine species currently belong to this genus – *Borniopsis tsurumaru*, *Borniopsis ariakensis*, *Borniopsis macrophthalmensis* (Morton & Scott, 1989), *Borniopsis maipoensis* (Morton & Scott, 1989), *Borniopsis nodosa* (Morton & Scott, 1989), *Borniopsis ochetostomae* (Morton & Scott, 1989), *Borniopsis subsinuata* (Lischke, 1871), *Borniopsis yamakawai* (Yokoyama, 1922) and *Borniopsis sagamiensis* (Habe, 1961) (Huber 2015). All of these species are known only from eastern Asia. Those *Borniopsis* species, for which the biology is known, are host-specific commensals associated with burrowing invertebrates (mantis shrimps, crabs, holothurians, sipunculans, echiurans and probably tanaids) (Kuroda 1937, Morton 1972, 1988, Morton and Scott 1989, Goto and Kato 2012, Goto et al. 2012). Host animals are different among the *Borniopsis* species, except for one species pair (*Borniopsis tsurumaru* and *Borniopsis ariakensis*), suggesting that this group diversified by repeated host shifts among various invertebrates in eastern Asia. This assumption is partially confirmed by molecular phylogeny (Goto et al. 2012).

In this study, we describe a new species of *Borniopsis*, which was collected from the synaptid sea cucumber *Patinapta ooplax* (von Marenzeller, 1881) on mud flats at the mouth of Souzu River, southwestern Shikoku Island, Japan. *Patinapta ooplax* is a small earthworm-like holothurian that burrows in muddy sediments in the intertidal zone. We compared its morphology and host associations with the other *Borniopsis* species. In addition, we reviewed galeommatoideans associated with apodid sea cucumbers.

## Materials and methods

An undescribed species of *Borniopsis* was found attached to the synaptid sea cucumber *Patinapta ooplax* in the mud flats at the mouth of the Souzu River, Ainan-cho, Ehime Prefecture, southwestern Shikoku Island, Japan (32°57'N, 132°33'E) on 20 May 2000. We collected the specimens of this bivalve species during spring low tides in 2000, 2001, 2003, 2006, 2007, 2009, 2012, and 2013. Seven specimens collected on 11 March 2012 and two specimens collected on 15 March 2013 were preserved in 100% ethanol and brought back to the laboratory and observed under a binocular dissecting microscope for description. One specimen collected on 12 April 2009 was bleached to remove the periostracum for observation of shell surface sculpture. We deposited the holotype and two paratypes in the Museum of Zoology, University of Michigan
(UMMZ), and two paratypes in National Museum of Nature and Science, Tokyo (NSMT). We also observed specimens of *Borniopsis tsurumaru* (SBMNH 149526), *Borniopsis ariakensis* (SBMNH35056), *Borniopsis ochetostomae* (SBMNH 149525) and *Borniopsis maipoensis* (SBMNH 35126) loaned from the Santa Barbara Natural History Museum. All the loaned specimens were collected from the mud flats of Hong Kong.

## Systematics

### Superfamily Galeommatoidea J.E. Gray, 1840 Family Galeommatidae *sensu lato* (Ponder, 1998) Genus *Borniopsis* Habe, 1959

#### Borniopsis mortoni sp. n.

Taxon classificationAnimaliaVeneroidaGaleommatidae

http://zoobank.org/1782E002-A7F1-42D1-A1EC-0824A89B5A3E

New Japanese name: Himoikarinamako-yadorigai

Figs 1 , 2 , 3 , 4

##### Material examined.

**Figure 1.**
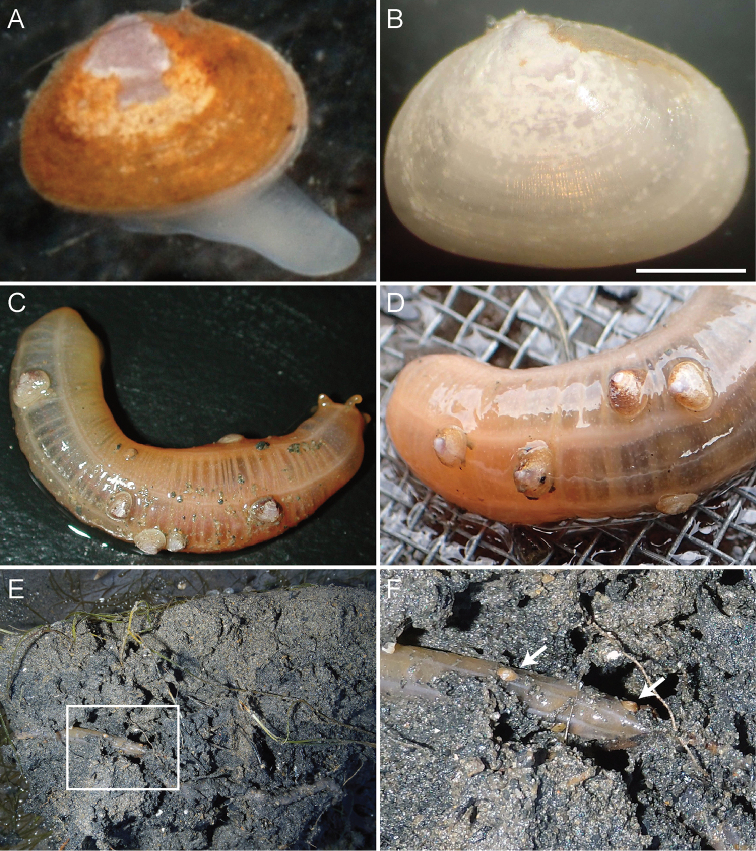
*Borniopsis mortoni* and its host. **A** A crawling individual of *Borniopsis mortoni*. **B** Right shell valve of Paratype 4 (NSMT Mo 78969) bleached to remove the periostracum for observation of shell surface sculpture **C, D** *Patinapta ooplax* with multiple individuals of *Borniopsis mortoni* attached **E, F** *Patinapta ooplax in situ* in mud flats with *Borniopsis mortoni* attached (arrowed). Photo credits: H. Ishikawa: **A–C, E, F**; Y. Hamamura: **D**. Scale bar: 1 mm.

**Figure 2.**
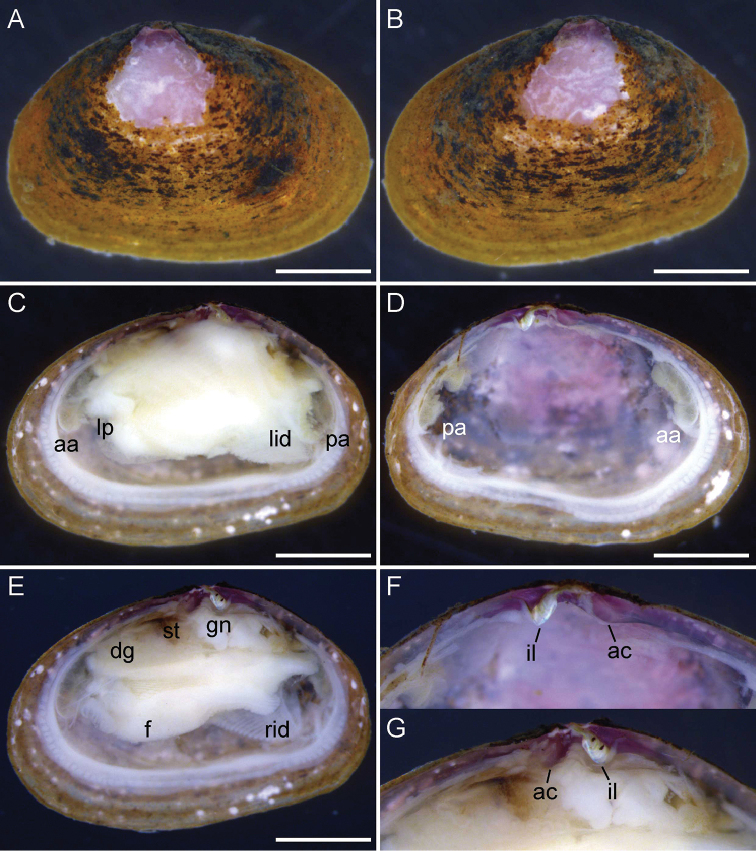
Holotype of *Borniopsis mortoni* (UMMZ 305035) (SL 4.1 mm). **A, C, E** Right valve **B, D** Left valve **F, G** Hinge structure of left and right valves. Abbreviations: aa, anterior adductor muscle; ac, anterior cardinal tooth; f, foot; gn, gonad; il, internal ligament; lid, inner demibranch of left side; pa, posterior adductor muscle; rid, inner demibranch of right side; st, stomach. Scale bars 1 mm. Photo credits: R. Goto: **A–G**.

**Figure 3.**
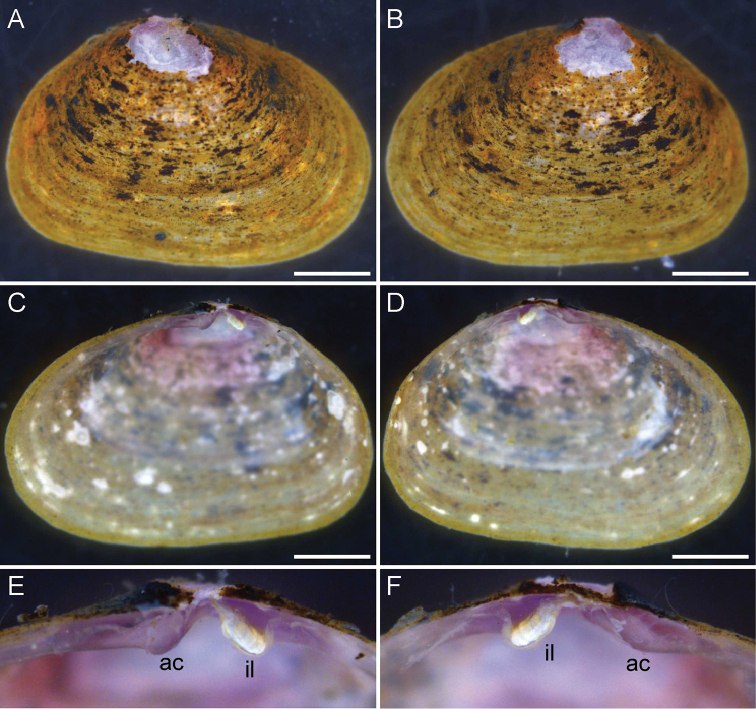
Paratype 1 of *Borniopsis mortoni* (UMMZ 305035) (SL 2.4 mm). **A, C** Right valve **B, D** Left valve **E, F** Hinge structure of right and left valves. Abbreviations: ac, anterior cardinal tooth; il, internal ligament. Scale bars 0.5 mm. Photo credits: R. Goto: **A–F**.

**Figure 4.**
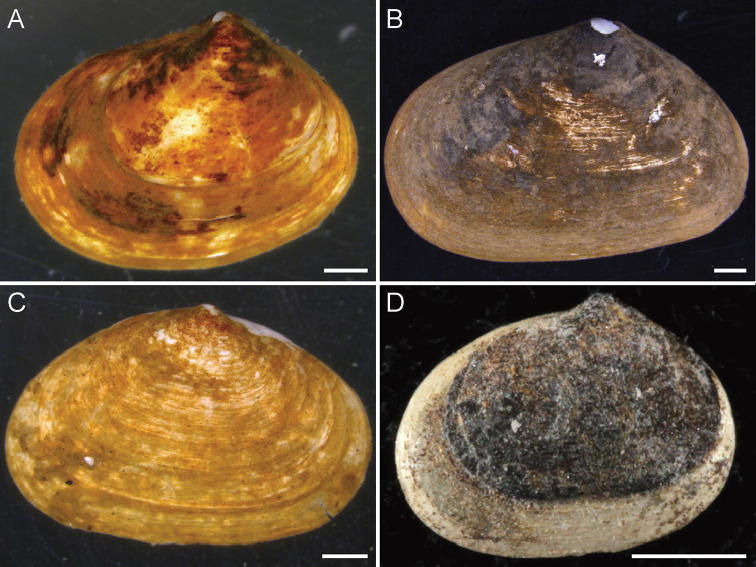
**A** *Borniopsis tsurumaru* (SBMNH 149526) **B** *Borniopsis ariakensis* (SBMNH 35056) **C** *Borniopsis ochetostomae* (SBMNH 149525) and **D** *Borniopsis maipoensis* (SBMNH 35126). Photo credits: R. Goto: **A–C**; P. Valentich-Scott: **D**. Scale bars 1 mm.

Holotype (Figs 1, 2): UMMZ 305035 (SL 4.1 mm, SH 2.8 mm). Paratype 1 (Fig. 3): UMMZ 305036-1 (SL 3.9 mm, SH 2.7 mm), paratype 2: UMMZ 30536-2 (SL 2.4 mm, SH 1.8 mm), paratype 3: NSMT-Mo 78968 (SL 3.7 mm, SH 2.7 mm) and paratype 4 (Fig. 1B): NSMT-Mo 78969 (SL 3.3 mm, SH 2.3 mm). Non-type specimens: four individuals of *Borniopsis mortoni* (SL 2.4, 3.3, 3.8, 3.3 mm). All specimens were collected in the mud flats at the mouth of the Souzu River, Ainan-cho, Ehime Prefecture, southwestern Shikoku Island, Japan (32°57'N, 132°33'E). Comparative species (Fig. 4): *Borniopsis tsurumaru*, SBMNH 149526; *Borniopsis ariakensis*, SBMNH 35056; *Borniopsis ochetostomae*, SBMNH149525; and *Borniopsis maipoensis*, SBMNH 35126.

##### Type locality and habitat.

Mud flats at the mouth of the Souzu River, Ainan-cho, Ehime, southwestern Shikoku Island, Japan (32°57'N, 132°33'E).

##### Description.

Shell (Figs 1–3): Shell small (up to 4.1 mm), thin, slightly inflated, shape elongate-ovate and equivalve; inequilateral, longer anteriorly. Anterior and posterior margins rounded, ventral margins slightly rounded. Umbo small. Beak small, prosogyrate, situated 2/3 of way toward posterior. Each valve covered by tan to dark brown periostracum with black deposits, often eroded around beaks (Figs 2, 3). Shell surface underneath periostracum smooth and whitish with pearly luster (Fig. 1B). Sculpture consisting of fine, dense and a few strong, widely spaced commarginal growth striae visible even on periostracum and very faint dense radial striae only visible underneath periostracum (Fig. 1B). Hinge of each valve consisting of a single stout cardinal tooth in front of umbo and well-developed oblique internal ligament posterior to umbo (Figs 2F, G; 3E, F). Soft parts (Figs 1, 2): Mantle not reflected, without prominent tentacles, edges narrowly extend beyond margin of shell, with numerous short papillae regularly arranged. Both anterior and posterior adductor muscles elongate-ovate, subequal and situated in the middle of dorsal and ventral margin. Ctenidia with gill axis nearly vertical, flat, consisting of single demibranch with both ascending and descending lamellae, joined anteriorly to inner and outer labial palps. Labial palps leaf-shaped. Foot slender, of moderate size, with small heel; byssal glands located just in front of heel. Gonads situated from middle to posterior in visceral mass just below umbo. Stomach and digestive gland large, occupying anterior part of visceral mass.

##### Distribution.

Only known from the type locality.

##### Host.

*Patinapta ooplax* (Echinodermata: Holothuroidea: Apodida: Synaptidae) (Fig. 1C–F).

##### Host association.

*Borniopsis mortoni* attaches to the body surface of *Patinapta ooplax* by both its foot and byssal threads (Fig. 1C–F). Individuals were patchily distributed in the mud flats. Within the particular patches we sampled, approximately 70% of synaptids served as hosts for *Borniopsis mortoni*. Several *Borniopsis mortoni* often occured on one the same host. At the maximum, more than 10 individuals were attached to a single host. Two *Patinapta ooplax* infested by *Borniopsis mortoni* also harbored the endoparasitic eulimid gastropod *Hypermastus lacteus* (A. Adams, 1864).

##### Etymology.

The specific name is dedicated to Dr. Brian Morton who has made great contributions to marine biology, marine ecology and malacology. He discovered many interesting commensal galeommatoidean species from Hong Kong, some of which now belong to the genus *Borniopsis*.

##### Remarks.

The genus *Borniopsis* has been variously assigned to the Kelliidae (Morton and Scott 1989), Lasaeidae (Bieler et al. 2010), and the subfamily Montacutinae within Galeommatidae *sensu lato* (Huber 2015). However, these family- or subfamily-level groupings are ill-defined when a range of characters and taxa are considered (Ponder 1998). Indeed, the molecular analyses conducted by Goto et al. (2012) showed that each of these groups is actually polyphyletic. In this study, we assigned the genus *Borniopsis* tentatively to Galeommatidae *sensu lato*, which Ponder (1998) defined by the same diagnosis that was applied to the superfamily Galeommatoidea, as did Huber (2015). Further taxonomic assignment of this genus within Galeommatoidea (or Galeommatidae *sensu lato*) should be delayed until its family-level (or subfamily-level) classification is revised.

As with *Borniopsis mortoni*, both *Borniopsis tsurumaru* and *Borniopsis ariakensis* have a symbiotic relationship with synaptid sea cucumbers (Morton 1988, Morton and Scott 1989, Lützen et al. 2004, Kai and Henmi 2008). However, the particular host species differ between them – *Patinapta ooplax* (host solely for *Borniopsis mortoni*) and *Protankyra bidentata* (Woodward & Barrett, 1858) (host for both *Borniopsis tsurumaru* and *Borniopsis ariakensis*) (Morton and Scott 1989, Lützen et al. 2004). *Borniopsis mortoni* always attaches directly onto the body surface of the host (this study), whereas *Borniopsis tsurumaru* can attach to the body surface of the host, or the wall of the host’s burrow, or to the carapace of commensal crabs living in the same burrows (Morton 1988, Morton and Scott 1989, Lützen et al. 2004, Kai and Henmi 2008, Goto et al. 2012). Furthermore, the number of bivalves per host is much higher in *Borniopsis mortoni* (several to more than 10) than *Borniopsis tsurumaru* and *Borniopsis ariakensis* (usually one) (Lützen et al. 2004, Goto, Ishikawa, and Hamamura, personal observations).

The shells of *Borniopsis tsurumaru* and *Borniopsis ariakensis* are much larger (up to 11–12 mm in SL) than those of *Borniopsis mortoni* (up to 4.1 mm) (Morton and Scott 1989) (Fig. 4A, B). Probably, this corresponds with the size of the host because *Protankyra bidentata* is much larger than *Patinapta ooplax*. The shells of *Borniopsis tsurumaru* and *Borniopsis ariakensis* are thicker and more inflated than those of *Borniopsis mortoni* (Lützen et al. 2004; this study). In addition, the shells of *Borniopsis mortoni* are always covered by a dark brown periostracum, whereas those of *Borniopsis tsurumaru* and *Borniopsis ariakensis* are often whitish, although some are dark brown. The umbones of *Borniopsis tsurumaru* and *Borniopsis ariakensis* are more protruding than those of *Borniopsis mortoni* (Fig. 4A, B). A molecular analysis is needed to understand whether these three synaptid-associated species are monophyletic or not. In addition, morphological variation of *Borniopsis tsurumaru* and *Borniopsis ariakensis* is apparently continuous (Goto, Ishikawa and Hamamura, pers. obs.) so molecular testing should be employed to investigate whether they can be distinguished genetically or not.

The present new species also closely resembles *Borniopsis ochetostomae* and *Borniopsis maipoensis* in having an elongate ovate shell covered by a brownish periostracum (Fig. 4C, D). However, *Borniopsis ochetostomae* is much larger (up to 11 mm) than *Borniopsis mortoni* and its beak is located more centrally than that of *Borniopsis mortoni* (Morton and Scott 1989, Jespersen et al. 2002, this study) (Fig. 4C). On the other hand, *Borniopsis maipoensis* is rather smaller (up to 3 mm) and more rounded than *Borniopsis mortoni* (Morton and Scott 1989, this study) (Fig. 4D). In addition, *Borniopsis maipoensis* has two distinct papillae on the dorsal surface of the foot (Morton and Scott 1989), whereas we did not observe such papillae on *Borniopsis mortoni* (Fig. 1A). And lastly, the hosts for these three species are different – *Borniopsis mortoni* (holothurian hosts), *Borniopsis ochetostomae* (echiuran hosts) and *Borniopsis maipoensis* (probably tanaid hosts) (Morton and Scott 1989, this study).

### Key to *Borniopsis*

**Table:** 

1	Beaks subcentral	**2**
–	Beaks near posterior end	**3**
2	Shell outline elliptical to subelliptical	**4**
–	Shell outline trigonal to subtrigonal	**5**
3	Shell outline subovate, without nodules on inner surface of anterior valve margin	**6**
–	Shell outline quadrate, with nodules on inner surface of anterior valve margin	***Borniopsis nodosa***
4	SH/SL 0.60–0.66 (Morton and Scott 1989)	***Borniopsis ochetostomae***
–	SH/SL 0.73 (this study)	***Borniopsis yamakawai***
5	Periostracum brownish, max. SL < 4 mm	***Borniopsis macrophthalmensis***
–	Periostracum whitish, max. SL > 4 mm	**7**
6	Umbo slightly protruding	**8**
–	Umbo not protruding, max. SL < 5 mm	***Borniopsis mortoni***
7	SH/SL 0.80 (Morton, 1972)	***Borniopsis subsinuata***
–	SH/SL 0.59–0.64 (Habe, 1961)	***Borniopsis sagamiensis***
8	With two distinctive papillae on dorsal surface of foot, max. SL < 3 mm	***Borniopsis maipoensis***
–	Without two distinctive papillae on dorsal surface of foot, max. SL > 3 mm	**9**
9	SH/SL 0.73 (this study)	***Borniopsis tsurumaru***
–	SH/SL 0.66 (this study)	***Borniopsis ariakensis***

## Discussion

Apodid sea cucumbers, including members of the families Synaptidae and Chiridotidae, are one of the major hosts for galeommatoideans (Boss 1965, Morton and Scott 1989, Kato 1998, Middelfart and Craig 2004) (Table 1). Including *Borniopsis mortoni*, at least 13 species are known to have commensal associations with apodid holothurians (Table 1). They can be separated into the following four groups: the first group includes *Anisodevonia*, *Austrodevonia*, *Devonia* and *Entovalva*, which have reduced shells covered by well-developed mantle lobes (Kawahara 1942, Kato 1998, Middelfart and Craig 2004); the second group includes *Borniopsis* covered in this study (Morton 1988, Morton and Scott 1989); the third groups includes *Montacuta* (Bateson 1923, Fox 1979); and the forth group includes *Scintillona* (Morton 1957, Ó Foighil and Gibson 1984). All of these four groups live attached to the apodid’s body surface, except for *Entovalva*, which lives inside the host’s oesophagus (Spärk 1931, Kato 1998). Molecular phylogenies suggested that associations with apodids have evolved repeatedly in the Galeommatoidea (Goto et al. 2012).

**Table 1.** Galeommatoidean bivalves commensal with apodid sea cucumbers.

Species	Host	Distribution	References
*Anisodevonia ohshimai* (Kawahara, 1942)	*Patinapta ooplax*	NW Pacific	Kawahara 1942; Kato 1998
*Austrodevonia sharnae* Middelfart & Craig, 2004	*Taeniogyrus australianus*	NW Pacific	Middelfart and Craig 2004
*Devonia perrieri* (Malard, 1903)	*Leptosynapta inhaerens*	NE Atlantic	Malard 1903
*Devonia semperi* (Oshima, 1930)	*Protankyra bidentata*	NW Pacific	Oshima 1930
*Devonia* sp.	*Protankyra similis*	NW Pacific	Semper 1868
*Entovalva amboinensis* (Spärck, 1931)	*Patinapta laevis*	NW Pacific	Spärck 1931
*Borniopsis tsurumaru* Habe, 1959	*Protankyra bidentata*	NW Pacific	Morton and Scott 1989
*Borniopsis ariakensis* Habe, 1959	*Protankyra bidentata*	NW Pacific	Morton and Scott 1989
*Borniopsis mortoni* sp. n.	*Patinapta ooplax*	NW Pacific	this study
*Montacuta donacina* (Wood, 1848)	*Leptosynapta inhaerens*	NE Atlantic	Bateson 1923
*Montacuta percompressa* Dall, 1899	*Leptosynapta tenuis*	NE Atlantic	Fox 1979
*Scintillona bellerophon* Ó Foighil & Gibson, 1984	*Leptosynapta clarki*	NE Pacific	Ó Foighil and Gibson 1984
*Scintillona zelandica* (Odhner 1924)	*Taeniogyrus dendyi*	New Zealand	Finlay 1927

*Borniopsis mortoni* lives commensally with the apodid sea cucumber *Patinapta ooplax* on the temperate coast of western Shikoku Island. On the other hand, another galeommatoidean species, *Anisodevonia ohshimai*, lives attached to *Patinapta ooplax* on the subtropical coast of the Ryukyu Islands, southwestern Japan (Kawahara 1942, Kato 1998). Extensive sampling for *Anisodevonia ohshimai* has been undertaken in the Ryukyu Islands (Kawahara 1942, Kato 1998, Kosuge 2001). However, *Borniopsis mortoni* has never been found there. Thus, *Borniopsis mortoni* probably does not occur on the subtropical coast of the Ryukyu Islands and may be restricted to more temperate coasts. An alternative hypothesis is that *Patinapta ooplax* used by *Anisodevonia ohshimai* in the Ryukyu Islands is a different species to that used by *Borniopsis mortoni* in eastern Shikoku Island. The taxonomy of this group of synaptid holothurians remains poorly understood and thus it is highly probable that *Patinapta ooplax* is a species complex.

A new species of the genus *Borniopsis* is described herein. This genus is restricted to eastern Asia, and each species apparently utilizes a different invertebrate host (Table 2), suggesting that its diversification was caused by repeated host shifting. The shell size varies among species (Table 2), which is probably a specialization to each particular commensal lifestyle. *Borniopsis macrophthalmensis*, *Borniopsis maipoensis* and *Borniopsis mortoni* are the three smallest species in this genus (Table 2). *Borniopsis macrophthalmensis* is attached to the body surface of highly-mobile intertidal crabs only by fine byssal threads (Morton and Scott 1989). If *Borniopsis macrophthalmensis* had a large, heavy shell, it would easily fall off the host crab when it scurries quickly on the surface of the mudflat. Thus, such a small shell is probably an adaptation for life on a highly-mobile crab. Similarly, another galeomatoidean species, *Arthritica japonica* Lützen & Takahashi, 2003, which is also attached to the body surface of crabs, has very small shells (up to 2.05 mm) (Lützen and Takahashi 2003). This may represent a morphological convergence to a similar commensal lifestyle and supports our hypothesis of reduction in shell size associated with fast-moving hosts mentioned above. On the other hand, the second species *Borniopsis maipoensis* lives commensally with the tanaid *Discapseudes* sp. (Morton and Scott 1989), whereas the third species *Borniopsis mortoni* lives with the synaptid sea cucumber *Patinapta ooplax*. The diameter of these host burrows is very small. Thus, the small-sized shells of *Borniopsis maipoensis* and *Borniopsis mortoni* are probably an adaptation to the mode of life in narrow host burrows.

The mudflats of eastern Asia evidently possess one of the richest burrowing invertebrate faunas in the world. However, burrow associates remain poorly understood in many of them. Thus, further investigation in this area could reveal increased diversity of this distinctive bivalve genus *Borniopsis*.

**Table 2.** *Borniopsis*, its host and maximum size (shell length).

Species	Host	Max. SL (mm)	References
*Borniopsis ariakensis* Habe, 1959	holothurian (*Protankyra bidentata*)	12.3	Habe 1959
*Borniopsis macrophthalmensis* (Morton & Scott 1989)	crab (*Macrophthalmus*)	3.2	Morton and Scott 1989
*Borniopsis maipoensis* (Morton & Scott 1989)	probably tanaid (*Discapseudes* sp.)	3.0	Morton and Scott 1989
*Borniopsis mortoni* sp. n.	holothurian (*Patinapta ooplax*)	4.1	this study
*Borniopsis nodosa* (Morton & Scott 1989)	sipunculan (*Sipunculus nudus*)	6.1	Morton and Scott 1989
*Borniopsis ochetostomae* (Morton & Scott 1989)	echiuran (*Listriolobus sorbillans*)	10.1	Morton and Scott 1989
*Borniopsis sagamiensis* (Habe 1961)	unknown	19.4	Habe 1961
*Borniopsis subsinuata* (Lischke, 1871)	mantis shrimp (*Squilla*, *Oratosquilla*)	12.0	Morton 1972
*Borniopsis tsurumaru* Habe, 1959	holothurian (*Protankyra bidentata*)	10.7	Habe 1959
*Borniopsis yamakawai* (Yokoyama, 1922)	echiuran (*Ochetostoma erythrogrammon*)	11.0	Goto and Kato 2012; this study

## Supplementary Material

XML Treatment for Borniopsis mortoni
